# Continuing and new roles for surgery in the management of patients with stage IV melanoma

**DOI:** 10.2217/mmt-2017-0024

**Published:** 2018-04-09

**Authors:** Erica B Friedman, John F Thompson

**Affiliations:** 1 Melanoma Institute Australia, The University of Sydney, Sydney NSW, Australia; 2 Sydney Medical School, The University of Sydney, Sydney NSW, Australia

**Keywords:** melanoma, metastases, multidisciplinary care, prognosis, surgery, systemic therapy

Until a few years ago, it was generally agreed that the best treatment option for melanoma patients with distant metastases (stage IV disease) was complete surgical resection, whenever possible. Those with more widespread disease or who were deemed unfit for surgery were referred to medical oncologists, but they had little to offer in the way of effective systemic therapy, and often simply recommended palliative end-of-life care.

In the second decade of the 21st century, however, we have witnessed a dramatic change in the management of metastatic melanoma, with the introduction of two novel therapeutic drug classes – targeted small molecule inhibitors of the oncogenic BRAF V600 mutation or a downstream signaling target (MEK), and immune checkpoint inhibitors consisting of monoclonal antibodies against CTLA-4 and PD-1.

Accordingly, clinical decision making for patients with stage IV melanoma has become increasingly complex, and multiple clinical trials are in progress to determine the best strategies to combine or sequence systemic treatment and surgery. Some believe that a complete paradigm shift in the approach to patients with metastatic melanoma has occurred, with surgeons no longer playing any useful role. A more enlightened view is that we have entered an era of truly integrated and carefully coordinated multidisciplinary care of these patients.

The reality is that surgery remains an excellent treatment option for patients with just one or a small number of distant metastases. Complete surgical resection offers a rapid, cost-effective means of rendering them clinically disease free and should be the first-line treatment in appropriately screened patients. This strategy is supported by the results of several clinical trials. Good survival outcomes were achieved in the Canvaxin™ stage IV trial, which compared patients who received adjuvant treatment with Bacillus Calmette–Guérin (BCG) and an allogenic melanoma vaccine after complete resection of metastatic disease to patients who received only BCG with placebo after resection [1]. While the study did not show any benefit in the vaccine-treated arm, 5-year overall survival (OS) rates following complete surgical resection were approximately 40% in both groups, substantially higher than would have been expected if the patients had been treated with the systemic therapies that were available at the time. The Southwestern Oncology Group's prospective multicenter trial of patients with surgically resectable metastatic melanoma also found that prolonged OS can be achieved by complete resection. While median relapse-free survival (RFS) was short (5 months), median OS was 21 months and 4-year survival was 31% [2]. In the first Multicenter Lymphadenectomy Trial, retrospective analysis of patients who developed distant metastases found that inclusion of surgery as part of the treatment plan conferred a survival advantage, even in patients who developed high-risk visceral metastases. If surgery was performed, the median survival was 15.8 months, with a 4-year survival of 20.8%, compared with 6.9 months and 7.0%, respectively, in patients who received systemic medical therapy alone (p < 0.0001; HR: 0.406) [3].

Not surprisingly, recurrence occurs in many patients after surgical resection of distant metastases [2,4]. However, resection continues to be a valuable option if further isolated recurrences develop; in one study of sequential complete resection for recurrences, a 19% 5-year survival rate was reported [5]. Stage IV patients in the first Multicenter Lymphadenectomy Trial cohort who had multiple resections had a median survival of 21 months and a 4-year survival of 25%, compared with 13 months and 18%, respectively, for patients who only had one operation (p = 0.0218) [3].

Appropriate selection of patients for surgical resection of oligometastatic disease is critically important, and only those who are medically fit for an operation, with anticipated low risks of operative morbidity and mortality, are suitable candidates. Minimally invasive operations, such as laparoscopic adrenalectomy for metastatic disease [6] and thoracoscopic resection of lung metastases [7], are now commonplace, decreasing morbidity and accelerating recovery time. Patients who present with single-organ-site metastases should be most strongly considered for surgery as first-line therapy, since the 5-year post-resection survival rate for patients with a solitary metastasis has been shown to be significantly better than for patients with four or more metastases (29 vs 11%; p < 0.0001) [8]. Sophisticated high-resolution imaging modalities are now readily available to better define the extent of metastatic disease and determine its resectibility [4]. Completely resected patients have an estimated 1-year OS of 75% (95% CI: 64–86%) compared with 25% (95% CI: 0–55%) after incomplete resection [2]. Finally, a long disease-free interval is predictive of significantly improved survival after surgical resection [3,8] and is another factor that should be considered when planning management.

How does surgical resection compare with results achieved by contemporary medical therapy? In BRAF-mutant patients, the combination of a BRAF-inhibitor (dabrafenib) and a MEK-inhibitor (trametinib) showed improved OS compared with BRAF inhibition alone, with a 72% OS rate at 12 months in the combination arm [9]. While the response rate was improved with dual therapy, the duration of response was still short (median 13.8 months) and in only 13% of patients was a complete response observed. Pooled analysis of 1861 patients with advanced melanoma treated with the anti-CTLA-4 antibody ipilimumab showed a 3-year survival rate of 22% [10]. This exceeded the 3-year survival rate of 12% for the best available conventional systemic therapy [11]. The PD-1 inhibitor pembrolizumab produced superior OS compared with ipilumumab, as well as the promise of more durable reponses [12]. The combination of ipilimumab and another PD-1 inhibitor, nivolumab, resulted in a progression-free survival of 11.5 months, longer than with either agent alone [13]. While this combination was associated with a 2-year survival rate of 79% in a Phase I trial, data from the Phase III trial are not yet sufficiently mature to determine if there will be a long-term survival benefit [13]. These promising results must be interpreted in the context of risk, which in the case of immunotherapy, is principally immune-related adverse events. The use of combination therapy brings with it a 55% rate of grade 3 or 4 adverse events [13], and some patients have long-term and occasionally fatal sequelae.

Inevitably, many patients will relapse after metastasectomy, and effective adjuvant postoperative therapies have long been sought. Recent data from two Phase III trials have demonstrated the value of adjuvant immunotherapy (nivolumab) and combination targeted therapy (dabrafenib plus trametinib), with significantly improved RFS in resected stage III/IV melanoma patients treated with these drugs [14,15]. In addition, resected stage III patients treated with combination targeted therapy had higher rates of OS compared with the placebo arm [14]. Patients are still likely to be candidates for systemic therapy with targeted agents or immunotherapy, even after multiple resections, if further relapse does occur.

Administration of systemic therapy prior to surgery for resectable disease (neoadjuvant therapy) has the theoretical benefit of treating any subclinical micrometastatic disease elsewhere, thus improving OS. It can also convert surgically unresectable disease to resectable disease, make the operation easier and less morbid, and provide information about tumor biology and response to treatment that may help to guide postoperative adjuvant treatment. Most of the limited available data on neoadjuvant therapy for metastatic melanoma are from patients with bulky stage III disease. Preoperative treatment with combination dabrafenib and trametinib has shown promise in such patients. In a small Phase II trial, six of 14 patients (43%) had a complete pathological complete response at the time of resection [16]. No patient's disease progressed during neoadjuvant treatment, however 86% developed side effects that interrupted treatment. In another trial comparing neoadjuvant combination targeted therapy to surgery followed by adjuvant therapy, which included patients with oligometastatic stage IV disease, similar results were observed. A pathological complete response was observed in 58% of patients and early analysis demonstrated a significant increase in RFS in the neoadjuvant arm [17]. However, in 92% of patients’ treatment was interrupted due to toxicity. In a Phase I trial of neoadjuvant ipilimumab and nivolumab in patients with palpable stage III melanoma, responses were frequently induced, but again at the cost of high toxicity, with early cessation of therapy in 18 of 20 patients [18].

A newly emerging role for the surgeon in the changing landscape of melanoma management is to obtain disease control while patients are receiving systemic therapy. For those receiving drug who have foci of progressive disease in the setting of otherwise regressing or stable disease, surgery can be a complementary treatment modality [19]. In patients who have had a good response but have limited residual disease that can be resected, metastasectomy can render a patient melanoma free. As well, surgery as a palliative measure should continue to be offered to patients with resectable metastases that are symptomatic, or likely to become symptomatic before demise from their disease.

In conclusion, while recent advances in the medical oncology arena are exciting and hold great promise, they do not provide a universally effective ‘magic bullet’ to treat patients with stage IV melanoma, and serious side effects are not uncommon. Surgery remains an important part of the therapeutic armamentarium and is likely to continue to have an important role for the foreseeable future. The optimal timing of surgery for stage IV melanoma, whether as first-line treatment, following neoadjuvant systemic treatment or as salvage therapy will continue to be an individually tailored decision for each patient, best made after discussion of that patient's situation by an appropriately constituted multidisciplinary team.
